# Crystal structure and Hirshfeld surface analysis of poly[[di-μ_3_-glycine-lithium] perchlorate]

**DOI:** 10.1107/S2056989018018145

**Published:** 2019-01-04

**Authors:** Palanisamy Revathi, Janani S. Mohan, Thangavelu Balakrishnan, Kandasamy Ramamurthi, Subbiah Thamotharan

**Affiliations:** a Crystal Growth Laboratory, PG and Research Department of Physics, Periyar EVR College (Autonomous), Tiruchirappalli 620 023, India; b Biomolecular Crystallography Laboratory, Department of Bioinformatics, School of Chemical and Biotechnology, SASTRA Deemed University, Thanjavur 613 401, India; c Department of Bio-Medical Engineering, Aarupadai Veedu Institute of Technology, Vinayaga Missions Research Foundation, Paiyanoor, Chennai 603 104, India

**Keywords:** crystal structure, zwitterionic glycine, C^α^—H⋯O inter­actions, glycine⋯perchlorate inter­action, distorted tetra­hedral geometry

## Abstract

In the title salt, {[Li(C_2_H_5_NO_2_)_2_]ClO_4_}_*n*_, the Li^+^ cation is coordinated by four carboxyl­ate oxygen atoms of the glycine mol­ecules with a distorted tetra­hedral geometry. The glycine exists in a zwitterionic form with protonated amino and deprotonated carboxyl­ate groups. In the crystalline state, the title salt is primarily stabilized by inter­molecular N—H⋯O and C^α^—H⋯O inter­actions which inter­connect various units. Hirshfeld surface analysis indicates that the inter­molecular H⋯O/O⋯H inter­actions are the most important contributors to the crystal packing.

## Abstract 2

Crystal and mol­ecular structure of bis­(glycinium)lithium perchlorate salt is reported and inter­molecular N— H⋯O and C—H⋯O hydrogen bonds stabilize the salt in the crystalline state.

## Chemical context   

As part of an ongoing effort aimed at the elucidation of the crystal and mol­ecular structures of several metal complexes/co-crystals originating from simple amino acids (Balakrishnan *et al.*, 2013*a* ▸,*b* ▸; Revathi *et al.*, 2015 ▸; Sathiskumar *et al.*, 2015*a* ▸,*b* ▸), we report herein the crystal structure of (I), a bis(glycinium)lithium perchlorate salt complex and discuss the hydrogen-bonding inter­actions it forms. The crystal packing and important molecular geometries of (I) are compared with a closely related structure bis(glycine)lithium nitrate salt complex (Baran *et al.*, 2009 ▸).
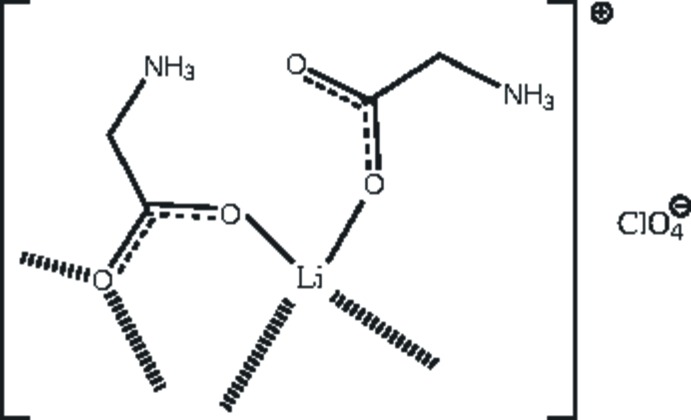


## Structural commentary   

**Figure 1:**
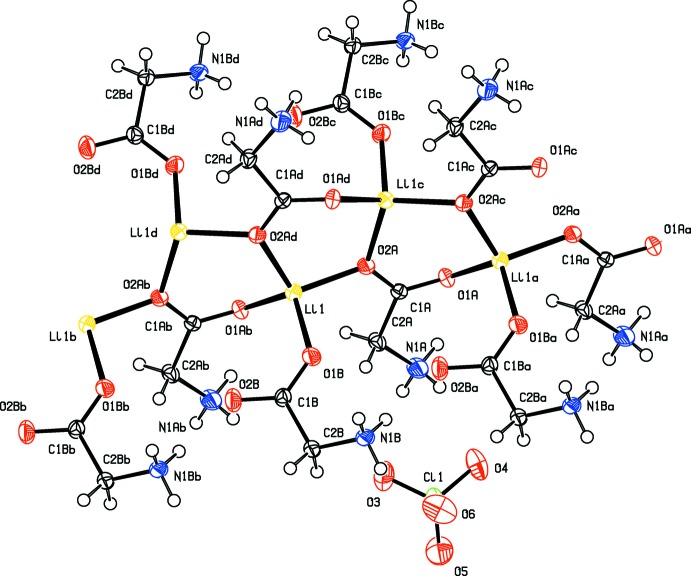
Part of the crystal structure of (I) showing the atomic labelling. Displacement ellipsoids are drawn at the 50% probability level. [Symmetry codes: (*a*) *x*, *y* − 1, *z*; (*b*) *x*, *y* + 1, *z*; (*c*) −*x* + 1, *y* − 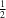, −*z* + 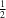; (*d*) −*x* + 1, *y* + 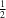, −*z* + 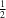].

An *ORTEP* view of the title salt is shown in Fig. 1 ▸. The asymmetric unit contains two glycinium units, one Li cation and a perchlorate anion. Both glycine mol­ecules exhibit a zwitterionic structure, as evident from the bond lengths involving the carboxyl­ate atoms (Table 1 ▸) and the protonation of the N atoms of the glycine mol­ecules. In (I), the torsion angle N1*A*—C2*A*—C1*A*—O1*A* in the one of the glycinium is −0.18 (19)°, while the corresponding angle is 20.75 (18)° in the other glycinium. The superposition of these two glycine mol­ecules involving non-hydrogen atoms reveals high degree of similarity with an r.m.s.d. value of 0.13 Å, the maximum deviation (0.19 Å) being observed at the *C* ^α^ (C2*A* and C2*B*) atom.

In the crystal, the Li cation is coordinated by four carboxyl­ate oxygen atoms of the glycine mol­ecules. One oxygen atom from each glycine mol­ecule is incorporated in the Li coordination sphere with Li—O distances ranging from 1.906 (3) to 2.015 (3) Å. The geometry around the Li cation is distorted tetra­hedral, as discernible from the angles around the Li cation (Table 1 ▸). The lithium coordination is extended as a layer that runs parallel to the *b* axis. The distance between two adjacent Li ions is 3.270 (13) Å.

In a closely related structure of the complex bis­(glycine) lithium nitrate (Baran *et al.*, 2009 ▸), the Li cation is surrounded by four carboxyl­ate oxygen atoms in a distorted tetra­hedral geometry as in (I). The distance between two adjacent Li ions is 5.034 Å.

## Supra­molecular features   

**Figure 2:**
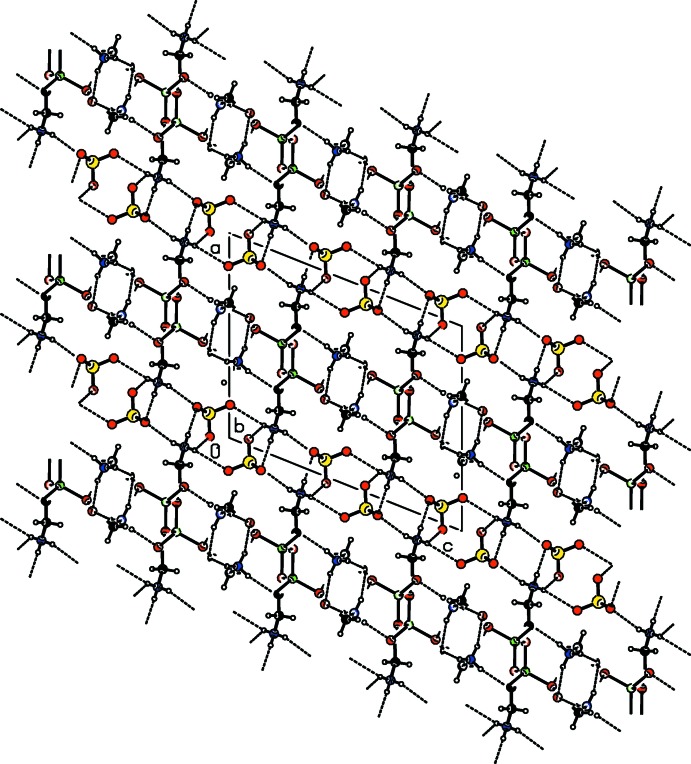
The crystal structure of (I), comprising alternate layers of Li–glycinium units and perchlorate anions, viewed down the *b* axis. Hydrogen bonds are indicated by dashed lines.

**Figure 3:**
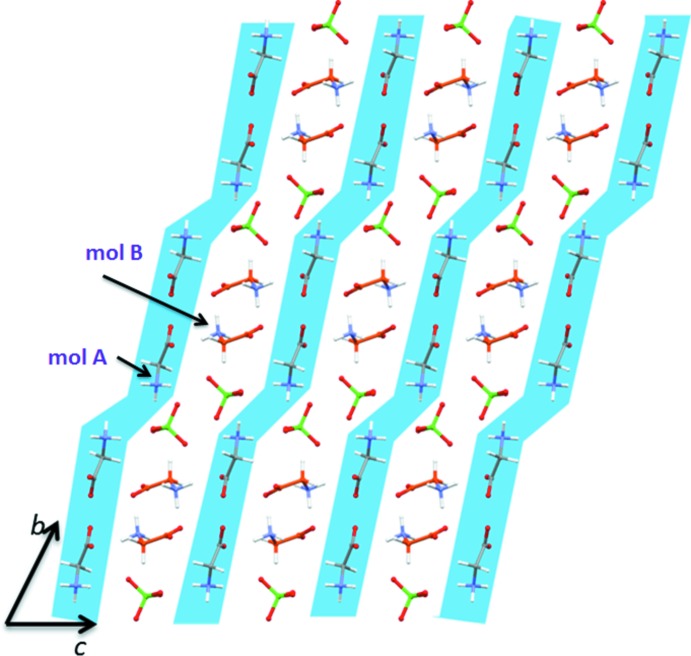
Second glycinium mol­ecules (orange) and perchlorate anions are sandwiched between arrays of the first glycinium mol­ecules (grey).

As shown in Table 2 ▸, the title salt is stabilized by a network of inter­molecular N—H⋯O, N—H⋯Cl and C^α^—H⋯O inter­actions. Overall, the crystal structure of the title salt can be described as alternate layers of perchlorate anions and Li-glycine cations (Fig. 2 ▸); these layers extend along the *c-*axis direction. In the crystalline state, each of the zwitterionic glycine mol­ecule is arranged in a different way. The first glycine, mol­ecule *A* (shown in grey), forms double arrays that run parallel to the *b*- and *c*-axis directions. In the array parallel to the *b* axis, the mol­ecules are oriented in opposite directions, as shown in Fig. 3 ▸. The first glycine mol­ecule also forms arrays running parallel to the *b* axis. The second glycine mol­ecules (shown in orange) and the perchlorate anions are sandwiched between adjacent arrays formed by the first glycinium mol­ecules (Fig. 3 ▸). Similar packing features are observed for bis­(glycine)lithium nitrate (Baran *et al.*, 2009 ▸).

**Figure 4:**
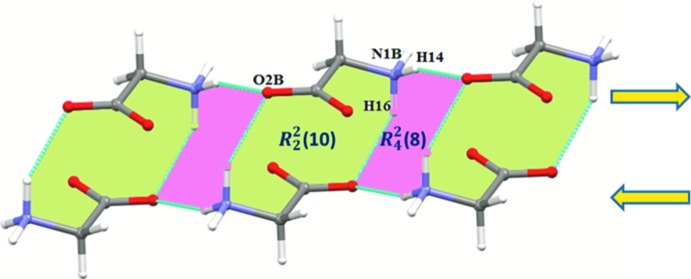
Anti-parallel glycinium arrays showing the formation of alternate 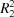
(10) and 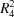
(8) motifs.

Furthermore, a careful examination of the crystal structure reveals that the first glycinium mol­ecule does not self-assemble in the solid state. It inter­acts with the perchlorate anion through inter­molecular N—H⋯O and N—H⋯Cl inter­actions and with the second glycinium via inter­molecular C^α^—H⋯O inter­actions. In contrast, the second glycinium mol­ecule is able to self-associate in the crystal through N—H⋯O inter­actions (involving H14⋯O2*B* and H16⋯O2*B*). The former linear hydrogen bond links the glycinium mol­ecules in a head-to-tail fashion in which amino acids are self-associated via their amino and carboxyl­ate groups. This is one of the characteristic features observed in many amino acids and amino acid complexes (Sharma *et al.*, 2006 ▸; Selvaraj *et al.*, 2007 ▸; Balakrishnan *et al.*, 2013*a* ▸,*b* ▸; Revathi *et al.*, 2015 ▸). Moreover, this head-to-tail chain sequence extends along the *b-*axis direction and adjacent chains are oriented in an anti-parallel fashion. Centrosymmetrically related dimers [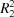
(10) motif] of the second glycinium mol­ecules are generated through H16⋯O2*B* inter­actions. Together, the H14⋯O2*B* and H16⋯O2*B* inter­actions lead to alternating 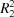
(10) and 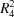
(8) motifs (Fig. 4 ▸).

**Figure 5:**
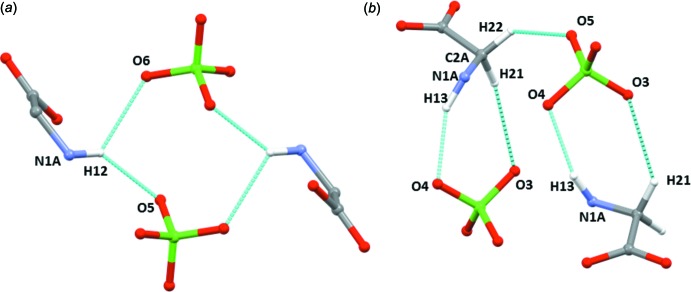
(*a*) The glycinium mol­ecules and perchlorate anions form a closed 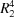
(8) loop through inter­molecular N—H⋯O hydrogen bonds. (*b*) The glycinium mol­ecules and perchlorate anions are inter­connected by N—H⋯O and C^α^—H⋯O inter­actions, forming a ring motif, with adjacent rings connected by C^α^—H⋯O inter­actions.

The protonated amino group of the first glycinium (mol *A*) is involved in five hydrogen-bonding (N—H⋯O and N—H⋯Cl) inter­actions (see Table 2 ▸). One of the bifurcated hydrogen-bonding inter­actions is formed between H12 and atoms O5 and O6 of the perchlorate anions. This inter­actions generate an 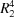
(8) loop motif in which two glycinium and two perchlorate ions are involved [Fig. 5 ▸(*a*)]. Inter­molecular N1*A*—H13⋯O4 and C2*A*—H21⋯O3 inter­actions connect the glycinium mol­ecules and perchlorate anions into a loop with adjacent loops being inter­connected by C2*A*—H22⋯O5 inter­actions [Fig. 5 ▸(*b*)]. As mentioned earlier, the second glycinium inter­acts with carboxyl­ate groups through its protonated amino group (N1*B*) (Fig. 4 ▸). It also inter­acts with the perchlorate anion through C2*B*—H24⋯O4 inter­action.

## Hirshfeld surface analysis and 2D fingerprint plots   

**Figure 6:**
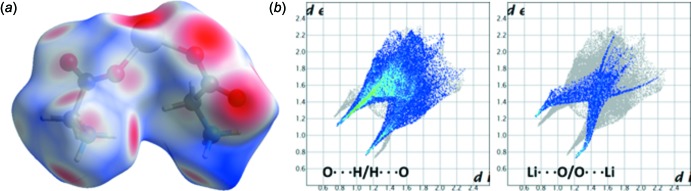
(*a*) Hirshfeld surface of the bis­(glycinium)lithium unit and (*b*) two-dimensional fingerprint plots for the inter­molecular O⋯H/H⋯O and Li⋯O/O⋯Li contacts.

The Hirshfeld surface (HS) analysis was carried out in order to understand the nature of the inter­molecular inter­actions present in the crystal structure. The shorter and longer contacts are indicated as red and blue spots on the HS and contacts with distances equal to the sum of the van der Waals radii are represented as white. The Hirshfeld surfaces for the cation (consisting of two glycinium mol­ecules and a lithium ion) and anion of the title salt complex were generated and analysed separately using the program *CrystalExplorer* (Wolff *et al.*, 2012 ▸). The HS of the cation mapped over the normalized distance, *d*_norm_, and the 2D fingerprint plots (Spackman & McKinnon, 2002 ▸) are illustrated in Fig. 6 ▸. In the cation, inter­molecular O⋯H/H⋯O inter­actions are predominant making a 66.9% contribution to the total HS. In the two-dimensional fingerprint plots, these contacts are depicted as a pair of sharp spikes with *d*_e_ + *d*_i_ ∼1.9 Å. There is a remarkable difference observed in the relative contribution of the H⋯O (donor region where *d*_e_ > *d*_i_) and O⋯H (acceptor region where *d*_e_ < *d*_i_) contacts. The former contact contributes 47.3%, while the contribution of the latter reciprocal contact is 19.6%. Similarly, the relative contribution of the Li⋯O/O⋯Li contacts is calculated to be 12.7% and these contacts appear as sharp spikes at a distance of around 1.9 Å. The proportions of Li⋯O and O⋯Li contacts are comparable (5.9 and 6.8%, respectively). The H⋯H contacts contribute 11.3% to the total HS of the cation part. The H⋯Li/Li⋯H (2.5%), O⋯C/C⋯O (2.4%) and O⋯O (2.0%) contacts play a minor role in the stabilization of the crystal structure.

**Figure 7:**
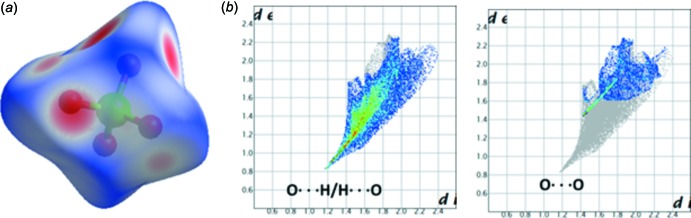
(*a*) Hirshfeld surface of the perchlorate unit and (*b*) two-dimensional fingerprint plots for the inter­molecular O⋯H/H⋯O and O⋯O contacts.

**Figure 8:**
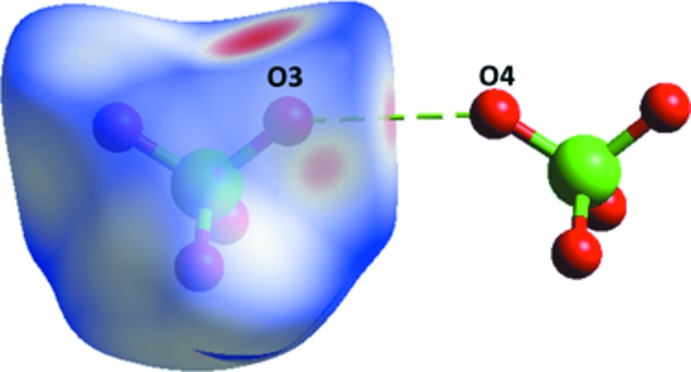
An inter­molecular O⋯O contact inter­connects adjacent percholorate anions.

In the perchlorate anion, the relative contributions of the O⋯H/H⋯O and O⋯O contacts are 81.4 and 18.2%, respectively (Fig. 7 ▸). The O⋯H/H⋯O contacts visible on the HS are due to the N—H⋯O and C—H⋯O hydrogen bonds. The O⋯O contacts are also visible on the HS and this contact of around 2.8 Å has the shortest distances of *d*_e_ and *d*_i_ of around 1.4 Å (Fig. 8 ▸). This O⋯O short contact [2.879 (2) Å] links the anionic mol­ecules into a chain running parallel to the *b-*axis direction.

## Database survey   

A search of the Cambridge Structural Database (CSD, version 5.39, last update August 2018; Groom *et al.*, 2016 ▸) using the keywords ‘lithium (name)’ and ‘amino-acids, peptides and complexes (class)’ yielded 50 hits of which 18 are glycine amino acids. In most of the complexes, carboxyl­ate oxygen atoms are involved in the Li coordination as described in the following examples. In the *catena*-[μ^3^-glycinato-*O*,*O*′)-(nitrato-*O*)lithium] complex, one of oxygen atoms of the nitro group is involved in the Li coordination along with the glycine carboxyl­ate O atoms (ALUNEA, Baran *et al.*, 2003 ▸). In three complexes (HEFWUK, Müller *et al.*, 1994 ▸; NEPWUC, Balakrishnan *et al.*, 2013*b* ▸; UCIYOV, Fleck *et al.*, 2006 ▸), the water O atom and three glycine carboxyl­ate O atoms partici­pate in the Li coordination. In the *catena*-[[μ^4^-glycyl-*N*,*O*,*O*,*O*′]lithium] complex (HEFXAR, Müller *et al.*, 1994 ▸), the deprotonated amino group N atom is involved in the Li coordination sphere and in cyclo­[tris­(glycyl-prolyl-*O*)]isothio­cyanato­lithium trihydrate (YUWXUJ, Thomas *et al.*, 1994 ▸), the N atom of the iso­thio­cynate, which acts as a fourth ligand, participates in the Li coordination sphere.

A detailed survey was also been conducted in the protein data bank (www.rcsb.org) to understand the Li^+^ coordination with protein mol­ecules. The keyword ‘lithium’ was used in the search, which resulted in 74 hits (up to 3.0 Å resolution). There are 30 structures found with better than 1.5 Å resolution and these structures were examined further. In this dataset, we found that Li is three- to six-coordinate with a water mol­ecule belonging to the Li complex cation. Moreover, the residues aspartate and glutamate are included in the Li coordination in several structures.

## Synthesis and crystallization   

The title salt was synthesized by dissolving AR-grade glycine and lithium perchlorate in a 2:1 stoichiometric ratio in double distilled water and stirred continuously for 2 h. Slow evaporation of this aqueous solution at room temperature yielded transparent colourless single crystals of the title salt.

## Refinement   

Crystal data, data collection and structure refinement details are summarized in Table 3 ▸. The positions of N-bound H atoms were located from a difference-Fourier map and refined freely along with their isotropic displacement parameters. The remaining H atoms were placed in calculated positions (C—H = 0.97 Å) and refined as riding with *U*_iso_(H) = 1.2*U*_eq_(C). Reflections 1 0 0 and 0 0 2 were obscured by the beam stop and were omitted during final refinement cycle.

## Supplementary Material

Crystal structure: contains datablock(s) I. DOI: 10.1107/S2056989018018145/ex2017sup1.cif

Structure factors: contains datablock(s) I. DOI: 10.1107/S2056989018018145/ex2017Isup2.hkl

CCDC reference: 1886641

Additional supporting information:  crystallographic information; 3D view; checkCIF report

## Figures and Tables

**Table 1:** Selected geometric parameters (Å, °)

O1*A*—Li1^i^	1.991 (3)	O2*A*—Li1	2.015 (3)
O2*A*—Li1^ii^	1.966 (3)	O1*B*—Li1	1.906 (3)
			
O1*B*—Li1—O1*A* ^iii^	108.95 (12)	O1*B*—Li1—O2*A*	102.15 (11)
O2*A* ^iv^—Li1—O1*A* ^iii^	102.03 (11)	O2*A* ^iv^—Li1—O2*A*	109.18 (12)

Symmetry codes: (i) 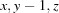; (ii) 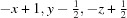; (iii) 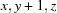; (iv) 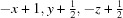.

**Table 2:** Hydrogen-bond geometry (Å, °)

*D*—H⋯*A*	*D*—H	H⋯*A*	*D*⋯*A*	*D*—H⋯*A*
N1*A*—H11⋯Cl1^v^	0.88 (3)	2.97 (3)	3.7635 (17)	151 (2)
N1*A*—H11⋯O4^v^	0.88 (3)	2.16 (3)	3.037 (2)	178 (2)
N1*A*—H12⋯O5^vi^	0.90 (3)	2.23 (3)	2.965 (2)	139 (2)
N1*A*—H12⋯O6^vii^	0.90 (3)	2.51 (3)	3.125 (2)	126 (2)
N1*A*—H13⋯O4	0.85 (3)	2.29 (3)	3.091 (2)	156 (2)
N1*B*—H14⋯O2*B* ^i^	0.90 (2)	2.00 (2)	2.8653 (19)	160.5 (18)
N1*B*—H15⋯O1*A* ^viii^	0.89 (2)	2.05 (2)	2.9308 (16)	169.8 (19)
N1*B*—H16⋯O1*B* ^ix^	0.93 (2)	2.35 (3)	2.9249 (17)	119 (2)
N1*B*—H16⋯O2*B* ^x^	0.93 (2)	2.28 (2)	3.119 (2)	150 (2)
C2*A*—H21⋯O1*B*	0.97	2.63	3.3747 (18)	134
C2*A*—H22⋯O5^v^	0.97	2.44	3.185 (2)	133
C2*B*—H23⋯O2*A* ^xi^	0.97	2.60	3.4867 (18)	152
C2*B*—H24⋯O4^iii^	0.97	2.55	3.360 (2)	141

Symmetry codes: (i) 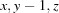; (iii) 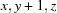; (v) 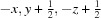; (vi) 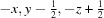; (vii) 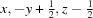; (viii) 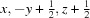; (ix) 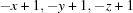; (x) 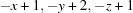; (xi) 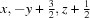.

**Table 3:** Experimental details

Crystal data	
Chemical formula	[Li(C_2_H_5_NO_2_)_2_]ClO_4_
*M*_r_	256.53
Crystal system, space group	Monoclinic, *P*2_1_/*c*
Temperature (K)	296
*a*, *b*, *c* (Å)	12.7792 (14), 5.2144 (4), 15.6368 (18)
β (°)	111.808 (4)
*V* (Å^3^)	967.40 (17)
*Z*	4
Radiation type	Mo *K*α
μ (mm^−1^)	0.43
Crystal size (mm)	0.35 × 0.30 × 0.30

Data collection	
Diffractometer	Bruker Kappa APEXII CCD
Absorption correction	Multi-scan (*SADABS*; Bruker, 2004 ▸)
*T*_min_, *T*_max_	0.865, 0.883
No. of measured, independent and observed [*I* > 2σ(*I*)] reflections	7318, 2304, 2047
*R*_int_	0.023
(sin θ/λ)_max_ (Å^−1^)	0.662

Refinement	
*R*[*F* ^2^ > 2σ(*F* ^2^)], *wR*(*F* ^2^), *S*	0.033, 0.092, 1.07
No. of reflections	2304
No. of parameters	170
H-atom treatment	H atoms treated by a mixture of independent and constrained refinement
Δρ_max_, Δρ_min_ (e Å^−3^)	0.52, −0.42

Computer programs: *APEX2*, *SAINT* and *XPREP* (Bruker, 2004 ▸), *SIR92* (Altomare *et al.*, 1994 ▸), *SHELXL2014/7* (Sheldrick, 2015 ▸), *PLATON* (Spek, 2009 ▸), *Mercury* (Macrae *et al.*, 2008 ▸) and *publCIF* (Westrip, 2010 ▸).

## supplementary crystallographic information

### Crystal data

**Table:**

[Li(C_2_H_5_NO_2_)_2_]ClO_4_	*F*(000) = 528
*M**_r_* = 256.53	*D*_x_ = 1.761 Mg m^−^^3^
Monoclinic, *P*2_1_/*c*	Mo *K*α radiation, λ = 0.71073 Å
*a* = 12.7792 (14) Å	Cell parameters from 4824 reflections
*b* = 5.2144 (4) Å	θ = 5.4–56.2°
*c* = 15.6368 (18) Å	µ = 0.43 mm^−^^1^
β = 111.808 (4)°	*T* = 296 K
*V* = 967.40 (17) Å^3^	Block, colourless
*Z* = 4	0.35 × 0.30 × 0.30 mm

### Data collection

**Table:**

Bruker Kappa APEXII CCD diffractometer	2047 reflections with *I* > 2σ(*I*)
ω and φ scan	*R*_int_ = 0.023
Absorption correction: multi-scan (SADABS; Bruker, 2004)	θ_max_ = 28.1°, θ_min_ = 3.4°
*T*_min_ = 0.865, *T*_max_ = 0.883	*h* = −16→16
7318 measured reflections	*k* = −6→4
2304 independent reflections	*l* = −20→20

### Refinement

**Table:**

Refinement on *F*^2^	Hydrogen site location: mixed
Least-squares matrix: full	H atoms treated by a mixture of independent and constrained refinement
*R*[*F*^2^ > 2σ(*F*^2^)] = 0.033	*w* = 1/[σ^2^(*F*_o_^2^) + (0.0468*P*)^2^ + 0.4555*P*] where *P* = (*F*_o_^2^ + 2*F*_c_^2^)/3
*wR*(*F*^2^) = 0.092	(Δ/σ)_max_ < 0.001
*S* = 1.07	Δρ_max_ = 0.52 e Å^−^^3^
2304 reflections	Δρ_min_ = −0.42 e Å^−^^3^
170 parameters	Extinction correction: SHELXL2014/7 (Sheldrick 2015), Fc^*^=kFc[1+0.001xFc^2^λ^3^/sin(2θ)]^-1/4^
0 restraints	Extinction coefficient: 0.051 (3)

### Special details

**Table:**

Geometry. All esds (except the esd in the dihedral angle between two l.s. planes) are estimated using the full covariance matrix. The cell esds are taken into account individually in the estimation of esds in distances, angles and torsion angles; correlations between esds in cell parameters are only used when they are defined by crystal symmetry. An approximate (isotropic) treatment of cell esds is used for estimating esds involving l.s. planes.

### Fractional atomic coordinates and isotropic or equivalent isotropic displacement parameters (Å^2^)

**Table:**

	*x*	*y*	*z*	*U*_iso_*/*U*_eq_	
O1A	0.32780 (8)	0.05574 (18)	0.19473 (6)	0.0197 (2)	
O2A	0.41860 (8)	0.43353 (19)	0.22629 (7)	0.0215 (2)	
O1B	0.41750 (10)	0.7653 (2)	0.38667 (7)	0.0268 (3)	
O2B	0.37499 (12)	1.1669 (2)	0.41178 (8)	0.0356 (3)	
N1A	0.13201 (12)	0.2513 (3)	0.19284 (12)	0.0312 (3)	
H11	0.068 (2)	0.335 (5)	0.1779 (16)	0.049 (6)*	
H12	0.128 (2)	0.138 (6)	0.1487 (19)	0.062 (7)*	
H13	0.135 (2)	0.163 (5)	0.2393 (19)	0.056 (7)*	
N1B	0.38884 (12)	0.5778 (3)	0.53719 (9)	0.0255 (3)	
H14	0.3687 (17)	0.465 (4)	0.4903 (16)	0.037 (5)*	
H15	0.3625 (17)	0.529 (4)	0.5801 (15)	0.037 (5)*	
H16	0.466 (2)	0.599 (5)	0.5661 (16)	0.051 (7)*	
C1A	0.33341 (11)	0.2912 (3)	0.21201 (8)	0.0157 (3)	
C1B	0.38168 (11)	0.9307 (3)	0.42732 (9)	0.0198 (3)	
C2A	0.22888 (11)	0.4280 (3)	0.21347 (10)	0.0224 (3)	
H21	0.2455	0.5039	0.2737	0.027*	
H22	0.2090	0.5653	0.1684	0.027*	
C2B	0.34142 (13)	0.8330 (3)	0.50229 (10)	0.0229 (3)	
H23	0.3636	0.9542	0.5530	0.027*	
H24	0.2598	0.8225	0.4775	0.027*	
Li1	0.4371 (2)	0.7982 (5)	0.27213 (16)	0.0196 (5)	
Cl1	0.07919 (3)	0.27547 (7)	0.41247 (3)	0.02800 (14)	
O3	0.09668 (13)	0.4960 (3)	0.36429 (11)	0.0504 (4)	
O4	0.08777 (12)	0.0478 (2)	0.36201 (10)	0.0453 (4)	
O5	−0.03202 (11)	0.2865 (3)	0.41610 (11)	0.0483 (4)	
O6	0.16270 (12)	0.2672 (3)	0.50378 (9)	0.0506 (4)	

### Atomic displacement parameters (Å^2^)

**Table:**

	*U*^11^	*U*^22^	*U*^33^	*U*^12^	*U*^13^	*U*^23^
O1A	0.0213 (5)	0.0155 (5)	0.0206 (5)	0.0022 (4)	0.0060 (4)	−0.0015 (4)
O2A	0.0183 (5)	0.0196 (5)	0.0296 (5)	−0.0016 (4)	0.0123 (4)	−0.0026 (4)
O1B	0.0422 (7)	0.0217 (5)	0.0228 (5)	0.0042 (4)	0.0192 (5)	0.0016 (4)
O2B	0.0639 (8)	0.0171 (5)	0.0338 (6)	0.0026 (5)	0.0275 (6)	0.0039 (4)
N1A	0.0194 (6)	0.0278 (7)	0.0493 (9)	−0.0026 (5)	0.0160 (6)	−0.0073 (7)
N1B	0.0357 (7)	0.0230 (7)	0.0248 (6)	0.0074 (5)	0.0192 (6)	0.0075 (5)
C1A	0.0161 (6)	0.0176 (6)	0.0129 (5)	0.0019 (5)	0.0050 (4)	0.0005 (4)
C1B	0.0256 (7)	0.0175 (7)	0.0167 (6)	−0.0007 (5)	0.0081 (5)	0.0006 (5)
C2A	0.0169 (6)	0.0182 (7)	0.0324 (7)	0.0002 (5)	0.0096 (5)	−0.0038 (5)
C2B	0.0312 (7)	0.0180 (7)	0.0246 (7)	0.0043 (5)	0.0163 (6)	0.0029 (5)
Li1	0.0217 (11)	0.0180 (11)	0.0206 (10)	0.0008 (9)	0.0096 (9)	0.0017 (9)
Cl1	0.0232 (2)	0.0203 (2)	0.0330 (2)	0.00037 (12)	0.00177 (15)	−0.00111 (13)
O3	0.0580 (9)	0.0278 (7)	0.0699 (10)	0.0008 (6)	0.0290 (8)	0.0097 (6)
O4	0.0509 (8)	0.0263 (7)	0.0500 (8)	0.0016 (5)	0.0085 (6)	−0.0107 (6)
O5	0.0259 (7)	0.0473 (8)	0.0679 (10)	−0.0025 (5)	0.0131 (6)	−0.0002 (7)
O6	0.0341 (7)	0.0704 (11)	0.0346 (7)	0.0116 (6)	−0.0019 (6)	−0.0082 (6)

### Geometric parameters (Å, º)

**Table:**

O1A—C1A	1.2535 (16)	N1B—H16	0.93 (2)
O1A—Li1^i^	1.991 (3)	C1A—C2A	1.5219 (18)
O2A—C1A	1.2670 (16)	C1B—C2B	1.5318 (19)
O2A—Li1^ii^	1.966 (3)	C2A—H21	0.9700
O2A—Li1	2.015 (3)	C2A—H22	0.9700
O1B—C1B	1.2543 (17)	C2B—H23	0.9700
O1B—Li1	1.906 (3)	C2B—H24	0.9700
O2B—C1B	1.2525 (18)	Li1—O2A^iii^	1.966 (3)
N1A—C2A	1.4793 (19)	Li1—O1A^iv^	1.991 (3)
N1A—H11	0.88 (3)	Li1—Li1^iii^	3.270 (3)
N1A—H12	0.90 (3)	Li1—Li1^ii^	3.270 (3)
N1A—H13	0.85 (3)	Cl1—O6	1.4308 (13)
N1B—C2B	1.4796 (19)	Cl1—O3	1.4371 (14)
N1B—H14	0.90 (2)	Cl1—O5	1.4443 (14)
N1B—H15	0.89 (2)	Cl1—O4	1.4521 (13)
			
C1A—O1A—Li1^i^	123.96 (11)	H21—C2A—H22	107.9
C1A—O2A—Li1^ii^	122.18 (11)	N1B—C2B—C1B	111.92 (12)
C1A—O2A—Li1	126.51 (11)	N1B—C2B—H23	109.2
Li1^ii^—O2A—Li1	110.42 (9)	C1B—C2B—H23	109.2
C1B—O1B—Li1	127.90 (12)	N1B—C2B—H24	109.2
C2A—N1A—H11	111.7 (17)	C1B—C2B—H24	109.2
C2A—N1A—H12	112.5 (17)	H23—C2B—H24	107.9
H11—N1A—H12	110 (2)	O1B—Li1—O2A^iii^	118.06 (13)
C2A—N1A—H13	112.8 (17)	O1B—Li1—O1A^iv^	108.95 (12)
H11—N1A—H13	104 (2)	O2A^iii^—Li1—O1A^iv^	102.03 (11)
H12—N1A—H13	106 (2)	O1B—Li1—O2A	102.15 (11)
C2B—N1B—H14	109.4 (13)	O2A^iii^—Li1—O2A	109.18 (12)
C2B—N1B—H15	108.6 (13)	O1A^iv^—Li1—O2A	117.22 (12)
H14—N1B—H15	110.6 (19)	O1B—Li1—Li1^iii^	121.15 (12)
C2B—N1B—H16	106.7 (16)	O2A^iii^—Li1—Li1^iii^	35.28 (8)
H14—N1B—H16	114 (2)	O1A^iv^—Li1—Li1^iii^	67.82 (6)
H15—N1B—H16	107.5 (19)	O2A—Li1—Li1^iii^	132.77 (14)
O1A—C1A—O2A	126.04 (12)	O1B—Li1—Li1^ii^	111.95 (11)
O1A—C1A—C2A	118.81 (12)	O2A^iii^—Li1—Li1^ii^	75.88 (12)
O2A—C1A—C2A	115.11 (12)	O1A^iv^—Li1—Li1^ii^	134.29 (14)
O2B—C1B—O1B	126.11 (13)	O2A—Li1—Li1^ii^	34.30 (3)
O2B—C1B—C2B	117.23 (13)	Li1^iii^—Li1—Li1^ii^	105.77 (13)
O1B—C1B—C2B	116.66 (12)	O6—Cl1—O3	110.10 (10)
N1A—C2A—C1A	111.90 (12)	O6—Cl1—O5	110.00 (9)
N1A—C2A—H21	109.2	O3—Cl1—O5	109.62 (9)
C1A—C2A—H21	109.2	O6—Cl1—O4	109.79 (8)
N1A—C2A—H22	109.2	O3—Cl1—O4	108.16 (9)
C1A—C2A—H22	109.2	O5—Cl1—O4	109.15 (8)
			
Li1^i^—O1A—C1A—O2A	53.01 (19)	Li1—O1B—C1B—O2B	−17.0 (2)
Li1^i^—O1A—C1A—C2A	−129.18 (14)	Li1—O1B—C1B—C2B	162.37 (14)
Li1^ii^—O2A—C1A—O1A	−2.3 (2)	O1A—C1A—C2A—N1A	−0.18 (19)
Li1—O2A—C1A—O1A	−170.40 (12)	O2A—C1A—C2A—N1A	177.86 (13)
Li1^ii^—O2A—C1A—C2A	179.83 (12)	O2B—C1B—C2B—N1B	−159.82 (14)
Li1—O2A—C1A—C2A	11.73 (18)	O1B—C1B—C2B—N1B	20.75 (18)

Symmetry codes: (i) *x*, *y*−1, *z*; (ii) −*x*+1, *y*−1/2, −*z*+1/2; (iii) −*x*+1, *y*+1/2, −*z*+1/2; (iv) *x*, *y*+1, *z*.

### Hydrogen-bond geometry (Å, º)

**Table:**

*D*—H···*A*	*D*—H	H···*A*	*D*···*A*	*D*—H···*A*
N1*A*—H11···Cl1^v^	0.88 (3)	2.97 (3)	3.7635 (17)	151 (2)
N1*A*—H11···O4^v^	0.88 (3)	2.16 (3)	3.037 (2)	178 (2)
N1*A*—H12···O5^vi^	0.90 (3)	2.23 (3)	2.965 (2)	139 (2)
N1*A*—H12···O6^vii^	0.90 (3)	2.51 (3)	3.125 (2)	126 (2)
N1*A*—H13···O4	0.85 (3)	2.29 (3)	3.091 (2)	156 (2)
N1*B*—H14···O2*B*^i^	0.90 (2)	2.00 (2)	2.8653 (19)	160.5 (18)
N1*B*—H15···O1*A*^viii^	0.89 (2)	2.05 (2)	2.9308 (16)	169.8 (19)
N1*B*—H16···O1*B*^ix^	0.93 (2)	2.35 (3)	2.9249 (17)	119 (2)
N1*B*—H16···O2*B*^x^	0.93 (2)	2.28 (2)	3.119 (2)	150 (2)
C2*A*—H21···O1*B*	0.97	2.63	3.3747 (18)	134
C2*A*—H22···O5^v^	0.97	2.44	3.185 (2)	133
C2*B*—H23···O2*A*^xi^	0.97	2.60	3.4867 (18)	152
C2*B*—H24···O4^iv^	0.97	2.55	3.360 (2)	141

Symmetry codes: (i) *x*, *y*−1, *z*; (iv) *x*, *y*+1, *z*; (v) −*x*, *y*+1/2, −*z*+1/2; (vi) −*x*, *y*−1/2, −*z*+1/2; (vii) *x*, −*y*+1/2, *z*−1/2; (viii) *x*, −*y*+1/2, *z*+1/2; (ix) −*x*+1, −*y*+1, −*z*+1; (x) −*x*+1, −*y*+2, −*z*+1; (xi) *x*, −*y*+3/2, *z*+1/2.
